# An ortho­rhom­bic polymorph of 1-benzyl-1*H*-benzimidazole

**DOI:** 10.1107/S1600536810015114

**Published:** 2010-04-28

**Authors:** Ying-jun Zhang, Xue-wen Zhu, Heng-yu Qian, Zhi-gang Yin, Chun-xia Zhang

**Affiliations:** a Key Laboratory of Surface and Interface Science of Henan, School of Materials and Chemical Engineering, Zhengzhou University of Light Industry, Zhengzhou 450002, People’s Republic of China

## Abstract

The title compound, C_14_H_12_N_2_, in contrast to the previously reported monoclinic polymorph [Lei *et al.* (2009 ▶). *Acta Cryst.* E**65**, o2613], crystallizes in the ortho­rhom­bic crystal system. The dihedral angle between the imidazole ring system and the phenyl ring is 76.78 (16)°. Weak C—H⋯N and C—H⋯π inter­actions are observed in the crystal structure.

## Related literature

For the synthesis, see: Lionel *et al.* (1996 ▶). For the monoclinic polymorph, see: Lei & Zhou (2009 ▶).
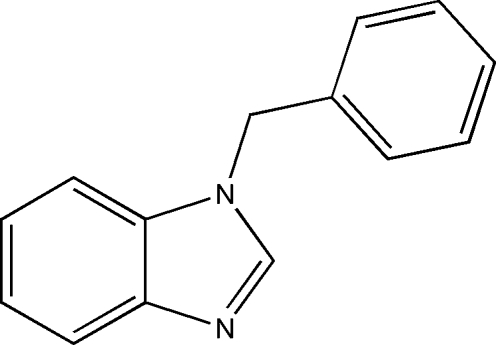


## Experimental

### 

#### Crystal data

C_14_H_12_N_2_
*M* *_r_* = 208.26
Orthorhombic, 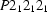

*a* = 6.124 (3) Å
*b* = 7.443 (4) Å
*c* = 23.860 (8) Å
*V* = 1087.6 (8) Å^3^
*Z* = 4
Mo *K*α radiation
μ = 0.08 mm^−1^
*T* = 293 K
0.25 × 0.20 × 0.18 mm

#### Data collection

Oxford Diffraction Xcalibur Eos Gemini diffractometer
3168 measured reflections
1886 independent reflections
892 reflections with *I* > 2σ(*I*)
*R*_int_ = 0.069

#### Refinement

*R*[*F* ^2^ > 2σ(*F* ^2^)] = 0.068
*wR*(*F* ^2^) = 0.148
*S* = 0.98
1886 reflections
154 parameters
H atoms treated by a mixture of independent and constrained refinement
Δρ_max_ = 0.19 e Å^−3^
Δρ_min_ = −0.20 e Å^−3^

### 

Data collection: *CrysAlis PRO* (Oxford Diffraction, 2008 ▶); cell refinement: *CrysAlis PRO*; data reduction: *CrysAlis PRO*; program(s) used to solve structure: *SHELXS97* (Sheldrick, 2008 ▶); program(s) used to refine structure: *SHELXL97* (Sheldrick, 2008 ▶); molecular graphics: *XP* in *SHELXTL* (Sheldrick, 2008 ▶); software used to prepare material for publication: *SHELXL97*.

## Supplementary Material

Crystal structure: contains datablocks I, global. DOI: 10.1107/S1600536810015114/hb5418sup1.cif

Structure factors: contains datablocks I. DOI: 10.1107/S1600536810015114/hb5418Isup2.hkl

Additional supplementary materials:  crystallographic information; 3D view; checkCIF report

## Figures and Tables

**Table 1:** Hydrogen-bond geometry (Å, °) *Cg*2 is the centroid of the C1–C6 ring.

*D*—H⋯*A*	*D*—H	H⋯*A*	*D*⋯*A*	*D*—H⋯*A*
C8—H8*B*⋯N2^i^	1.03 (5)	2.55 (5)	3.570 (8)	171 (4)
C12—H12⋯*Cg*2^ii^	0.93	2.66	3.559 (5)	162

Symmetry codes: (i) 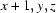; (ii) 
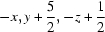
.

**Fig. 1.**
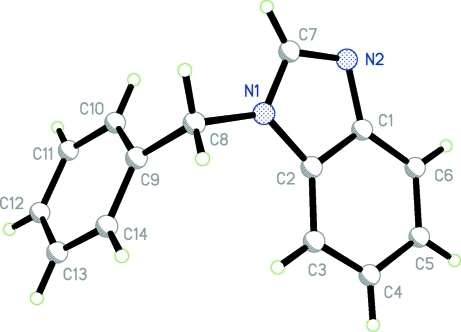
The asymmetric unit of (I), showing 50% probability displacement ellipsoids.

**Fig. 2.**
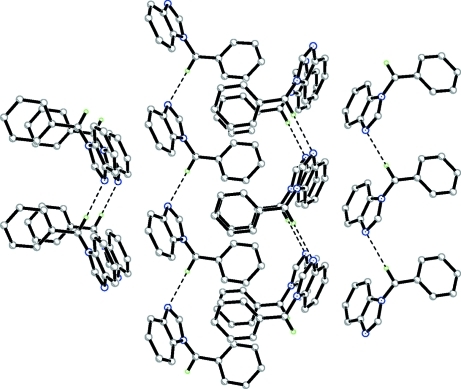
The crystal packing of (I), showing the hydrogen-bonded (dashed lines) network. H atoms not involved in the hydrogen bond interactions have been omitted for clarity.

## supplementary crystallographic information

### Comment

The title compound, 1-benzyl-1*H*-benzimidazole was first synthesized by Lionel (Lionel *et al.* 1996) using DMF as solvent.

The structure reported here is an orthorhombic form polymorph of the title compound, (C_14_H_12_N_2_), which has been characterized previously in a monoclinic form (Lei & Zhou, 2009). The bond lengths and angles are closely similar to those previously described . The dihedral angle between the imidazole ring and the benzyl ring is 76.78 (16)°, indicated that those two rings are not mutually perpendicular. In the crystal structure, molecules are linked *via* weak intermolecular C—H···N interactions, forming a chain along the *b*-axis direction. The structure is further stabilized by C—H···π contacts involving both of the aromatic rings. This arrangement is similar to that observed in the monoclinic polymorph.

### Experimental

The title compound was obtained by reacting benzimidazole (1.18 g, 0.01 mol) with benzyl chloride (1.25 g, 0.01 mol) and potassium carbonate (1.38 g, 0.01 mol) in acetone (50 ml). The reaction mixture was refluxed for 10 h. After removal of the solvents, the residue was dispersed in water to obtain an oil layer. Then the oil was dissolved in hot ethanol/water (2:1) and colourless blocks of (I) arose.

### Refinement

The absolute sturcture of (I) is indeterminate in the present refinement.  The methylene H atoms were freely refined. The other H atoms were positioned geometrically (C—H = 0.93Å) and refined as riding with *U*iso(H) = 1.2*U*eq(C).

### Crystal data

**Table:**

C_14_H_12_N_2_	*D*_x_ = 1.272 Mg m^−^^3^
*M**_r_* = 208.26	Melting point: 387 K
Orthorhombic, *P*2_1_2_1_2_1_	Mo *K*α radiation, λ = 0.71073 Å
Hall symbol: P 2ac 2ab	Cell parameters from 500 reflections
*a* = 6.124 (3) Å	θ = 3.2–26.2°
*b* = 7.443 (4) Å	µ = 0.08 mm^−^^1^
*c* = 23.860 (8) Å	*T* = 293 K
*V* = 1087.6 (8) Å^3^	Block, colorless
*Z* = 4	0.25 × 0.20 × 0.18 mm
*F*(000) = 440	

### Data collection

**Table:**

Oxford Diffraction Xcalibur Eos Gemini diffractometer	*R*_int_ = 0.069
Radiation source: fine-focus sealed tube	θ_max_ = 26.2°, θ_min_ = 3.2°
graphite	*h* = −7→7
φ and ω scans	*k* = −9→6
3168 measured reflections	*l* = −22→29
1886 independent reflections	1886 standard reflections every 0 min
892 reflections with *I* > 2σ(*I*)	intensity decay: none

### Refinement

**Table:**

Refinement on *F*^2^	Secondary atom site location: difference Fourier map
Least-squares matrix: full	Hydrogen site location: inferred from neighbouring sites
*R*[*F*^2^ > 2σ(*F*^2^)] = 0.068	H atoms treated by a mixture of independent and constrained refinement
*wR*(*F*^2^) = 0.148	*w* = 1/[σ^2^(*F*_o_^2^) + (0.0605*P*)^2^] where *P* = (*F*_o_^2^ + 2*F*_c_^2^)/3
*S* = 0.98	(Δ/σ)_max_ < 0.001
1886 reflections	Δρ_max_ = 0.19 e Å^−^^3^
154 parameters	Δρ_min_ = −0.20 e Å^−^^3^
0 restraints	Extinction correction: *SHELXL97* (Sheldrick, 2008), Fc^*^=kFc[1+0.001xFc^2^λ^3^/sin(2θ)]^-1/4^
Primary atom site location: structure-invariant direct methods	Extinction coefficient: 0.022 (5)

### Special details

**Table:**

Geometry. All esds (except the esd in the dihedral angle between two l.s. planes) are estimated using the full covariance matrix. The cell esds are taken into account individually in the estimation of esds in distances, angles and torsion angles; correlations between esds in cell parameters are only used when they are defined by crystal symmetry. An approximate (isotropic) treatment of cell esds is used for estimating esds involving l.s. planes.
Refinement. Refinement of *F*^2^ against ALL reflections. The weighted *R*-factor wR and goodness of fit *S* are based on *F*^2^, conventional *R*-factors *R* are based on *F*, with *F* set to zero for negative *F*^2^. The threshold expression of *F*^2^ > σ(*F*^2^) is used only for calculating *R*-factors(gt) etc. and is not relevant to the choice of reflections for refinement. *R*-factors based on *F*^2^ are statistically about twice as large as those based on *F*, and *R*- factors based on ALL data will be even larger.

### Fractional atomic coordinates and isotropic or equivalent isotropic displacement parameters (Å^2^)

**Table:**

	*x*	*y*	*z*	*U*_iso_*/*U*_eq_	
N1	0.9512 (6)	0.9532 (5)	0.13981 (13)	0.0379 (9)	
C2	0.9503 (7)	1.1255 (6)	0.16196 (16)	0.0376 (11)	
C7	0.7567 (8)	0.8802 (8)	0.15157 (18)	0.0515 (13)	
H7	0.7190	0.7639	0.1411	0.062*	
N2	0.6248 (7)	0.9861 (7)	0.17888 (16)	0.0614 (13)	
C3	1.1077 (8)	1.2581 (7)	0.16401 (18)	0.0524 (14)	
H3	1.2438	1.2429	0.1474	0.063*	
C10	0.9963 (8)	0.9160 (6)	0.01210 (18)	0.0463 (13)	
H10	0.8689	0.8567	0.0224	0.056*	
C9	1.1566 (7)	0.9393 (6)	0.05047 (16)	0.0379 (11)	
C14	1.3448 (8)	1.0270 (7)	0.03496 (18)	0.0478 (13)	
H14	1.4553	1.0447	0.0611	0.057*	
C8	1.1293 (10)	0.8674 (8)	0.1093 (2)	0.0502 (13)	
C13	1.3702 (9)	1.0881 (7)	−0.0187 (2)	0.0567 (15)	
H13	1.4984	1.1465	−0.0290	0.068*	
C11	1.0182 (9)	0.9782 (7)	−0.04190 (18)	0.0564 (14)	
H11	0.9057	0.9624	−0.0676	0.068*	
C1	0.7441 (8)	1.1405 (8)	0.18669 (18)	0.0484 (14)	
C5	0.8491 (14)	1.4320 (9)	0.2169 (2)	0.0766 (19)	
H5	0.8180	1.5377	0.2362	0.092*	
C4	1.0542 (12)	1.4117 (8)	0.1914 (2)	0.0692 (17)	
H4	1.1550	1.5049	0.1934	0.083*	
C12	1.2063 (9)	1.0635 (6)	−0.05752 (19)	0.0570 (15)	
H12	1.2236	1.1045	−0.0941	0.068*	
C6	0.6953 (10)	1.3013 (9)	0.21424 (19)	0.0647 (17)	
H6	0.5588	1.3184	0.2305	0.078*	
H8B	1.274 (8)	0.887 (6)	0.1303 (18)	0.058 (15)*	
H8A	1.078 (7)	0.746 (6)	0.1111 (16)	0.046 (13)*	

### Atomic displacement parameters (Å^2^)

**Table:**

	*U*^11^	*U*^22^	*U*^33^	*U*^12^	*U*^13^	*U*^23^
N1	0.037 (2)	0.043 (2)	0.0343 (18)	−0.002 (2)	0.0042 (18)	0.0001 (19)
C2	0.043 (3)	0.047 (3)	0.023 (2)	0.001 (3)	−0.011 (2)	0.000 (2)
C7	0.045 (3)	0.057 (3)	0.052 (3)	−0.010 (3)	0.000 (3)	0.009 (3)
N2	0.051 (3)	0.081 (3)	0.052 (3)	0.001 (3)	0.008 (2)	−0.001 (3)
C3	0.059 (3)	0.051 (3)	0.048 (3)	−0.005 (3)	−0.017 (3)	0.004 (3)
C10	0.051 (3)	0.046 (3)	0.042 (2)	−0.013 (3)	0.005 (3)	−0.007 (2)
C9	0.039 (3)	0.038 (3)	0.037 (2)	0.003 (3)	−0.001 (3)	−0.011 (2)
C14	0.041 (3)	0.054 (3)	0.049 (3)	−0.003 (3)	−0.002 (3)	−0.007 (3)
C8	0.053 (3)	0.052 (3)	0.045 (3)	0.007 (3)	0.004 (3)	0.005 (3)
C13	0.054 (3)	0.053 (4)	0.063 (3)	−0.007 (3)	0.022 (3)	−0.006 (3)
C11	0.064 (3)	0.061 (3)	0.044 (3)	−0.004 (3)	−0.008 (3)	−0.003 (3)
C1	0.048 (3)	0.072 (4)	0.025 (2)	0.021 (3)	0.007 (3)	0.006 (3)
C5	0.119 (6)	0.069 (4)	0.042 (3)	0.032 (5)	−0.038 (4)	−0.026 (3)
C4	0.095 (5)	0.053 (4)	0.060 (3)	0.003 (3)	−0.025 (4)	−0.006 (3)
C12	0.078 (4)	0.048 (3)	0.045 (3)	0.003 (3)	0.016 (3)	−0.003 (3)
C6	0.072 (4)	0.090 (5)	0.032 (3)	0.029 (4)	0.002 (3)	−0.010 (3)

### Geometric parameters (Å, °)

**Table:**

N1—C7	1.339 (6)	C14—C13	1.367 (6)
N1—C2	1.387 (5)	C14—H14	0.9300
N1—C8	1.459 (6)	C8—H8B	1.03 (5)
C2—C3	1.380 (6)	C8—H8A	0.96 (5)
C2—C1	1.398 (6)	C13—C12	1.379 (6)
C7—N2	1.303 (6)	C13—H13	0.9300
C7—H7	0.9300	C11—C12	1.367 (6)
N2—C1	1.374 (6)	C11—H11	0.9300
C3—C4	1.358 (7)	C1—C6	1.398 (7)
C3—H3	0.9300	C5—C6	1.356 (8)
C10—C9	1.354 (5)	C5—C4	1.404 (8)
C10—C11	1.376 (6)	C5—H5	0.9300
C10—H10	0.9300	C4—H4	0.9300
C9—C14	1.375 (6)	C12—H12	0.9300
C9—C8	1.511 (6)	C6—H6	0.9300
			
C7—N1—C2	107.0 (4)	N1—C8—H8A	98 (3)
C7—N1—C8	126.3 (4)	C9—C8—H8A	114 (2)
C2—N1—C8	126.7 (4)	H8B—C8—H8A	113 (4)
C3—C2—N1	132.2 (4)	C14—C13—C12	120.1 (5)
C3—C2—C1	124.0 (5)	C14—C13—H13	119.9
N1—C2—C1	103.8 (4)	C12—C13—H13	119.9
N2—C7—N1	114.3 (5)	C12—C11—C10	119.6 (5)
N2—C7—H7	122.9	C12—C11—H11	120.2
N1—C7—H7	122.9	C10—C11—H11	120.2
C7—N2—C1	104.1 (4)	N2—C1—C6	131.7 (5)
C4—C3—C2	116.8 (5)	N2—C1—C2	110.9 (4)
C4—C3—H3	121.6	C6—C1—C2	117.4 (6)
C2—C3—H3	121.6	C6—C5—C4	121.6 (5)
C9—C10—C11	121.3 (5)	C6—C5—H5	119.2
C9—C10—H10	119.4	C4—C5—H5	119.2
C11—C10—H10	119.4	C3—C4—C5	121.1 (6)
C10—C9—C14	119.1 (4)	C3—C4—H4	119.5
C10—C9—C8	120.2 (5)	C5—C4—H4	119.5
C14—C9—C8	120.7 (4)	C11—C12—C13	119.5 (4)
C13—C14—C9	120.4 (5)	C11—C12—H12	120.3
C13—C14—H14	119.8	C13—C12—H12	120.3
C9—C14—H14	119.8	C5—C6—C1	119.2 (6)
N1—C8—C9	113.0 (4)	C5—C6—H6	120.4
N1—C8—H8B	110 (2)	C1—C6—H6	120.4
C9—C8—H8B	108 (2)		
			
C7—N1—C2—C3	177.5 (5)	C14—C9—C8—N1	116.5 (6)
C8—N1—C2—C3	−2.7 (7)	C9—C14—C13—C12	0.5 (8)
C7—N1—C2—C1	0.4 (4)	C9—C10—C11—C12	0.8 (7)
C8—N1—C2—C1	−179.8 (4)	C7—N2—C1—C6	−178.1 (4)
C2—N1—C7—N2	0.5 (5)	C7—N2—C1—C2	1.5 (5)
C8—N1—C7—N2	−179.2 (4)	C3—C2—C1—N2	−178.6 (4)
N1—C7—N2—C1	−1.2 (5)	N1—C2—C1—N2	−1.2 (5)
N1—C2—C3—C4	−177.3 (4)	C3—C2—C1—C6	1.1 (6)
C1—C2—C3—C4	−0.7 (7)	N1—C2—C1—C6	178.5 (4)
C11—C10—C9—C14	0.0 (7)	C2—C3—C4—C5	0.8 (7)
C11—C10—C9—C8	−179.2 (5)	C6—C5—C4—C3	−1.4 (8)
C10—C9—C14—C13	−0.6 (7)	C10—C11—C12—C13	−0.9 (7)
C8—C9—C14—C13	178.5 (5)	C14—C13—C12—C11	0.3 (7)
C7—N1—C8—C9	106.1 (5)	C4—C5—C6—C1	1.8 (8)
C2—N1—C8—C9	−73.7 (6)	N2—C1—C6—C5	178.0 (5)
C10—C9—C8—N1	−64.3 (6)	C2—C1—C6—C5	−1.6 (7)

### Hydrogen-bond geometry (Å, °)

**Table:**

Cg2 is the centroid of the C1–C6 ring.

**Table:**

*D*—H···*A*	*D*—H	H···*A*	*D*···*A*	*D*—H···*A*
C8—H8B···N2^i^	1.03 (5)	2.55 (5)	3.570 (8)	171 (4)
C12—H12···Cg2^ii^	0.93	2.66	3.559 (5)	162

Symmetry codes: (i) *x*+1, *y*, *z*; (ii) −*x*, *y*+5/2, −*z*+1/2.
